# Codon usage bias analysis of the *WRKY* gene family in *Musa acuminata*

**DOI:** 10.3389/fgene.2025.1647037

**Published:** 2025-08-29

**Authors:** Jiaman Sun, Ji Zhang, Jinzhong Zhang, Brett J. Ferguson, Andrew Chen

**Affiliations:** ^1^ School of Life Science, Jiaying University, Meizhou, Guangdong, China; ^2^ Guangxi Academy of Agricultural Sciences, Nanning, China; ^3^ Guangzhou Academy of Agricultural and Rural Sciences, Guangzhou, China; ^4^ Integrative Legume Research Group, School of Agriculture and Food Sustainability, The University of Queensland, Brisbane, QLD, Australia; ^5^ School of Agriculture and Food Sustainability, The University of Queensland, Brisbane, QLD, Australia

**Keywords:** Musa acuminata, ‘Guijiao 9′, WRKY transcription factors, codon usage bias, natural selection, mutation pressure, Fusarium wilt of banana

## Abstract

Codon usage bias (CUB), a universal evolutionary phenomenon, reflects selective pressures shaping genome adaptation. The *WRKY* transcription factor family plays a pivotal role in regulating plant responses to physiological and biochemical stresses. This study investigates CUB patterns in 151 *WRKY* transcription factors of *Musa acuminata* ‘Guijiao 9′, a banana cultivar exhibiting resistance to Fusarium wilt Tropical Race 4 (TR4), to elucidate evolutionary drivers of stress adaptation. The codons of these transcription factors were selected based on their expression from RNA-Seq data. The average GC content of *MaWRKY* genes was 56.55%, with a GC3 content of 62.23%, indicating a preference for G/C-ending codons. Among the codons, 26 were identified as high frequency, with 22 ending in G or C. The high effective number of codons (ENC) values (35.03–60.14) suggested weak CUB. ENC-plot, PR2 bias plot, and neutrality analysis revealed that both natural selection and mutation pressure contributed to the observed CUB, with natural selection being the dominant factor influencing the codon usage of *MaWRKY* genes in *M*. *acuminata* ‘Guijiao 9'. Fifteen optimal codons, all ending in G or C, were identified. This analysis provides a theoretical foundation for further understanding the evolutionary mechanisms of *WRKY* genes in *Musa*.

## 1 Introduction

A codon is a triad nucleotide sequence on messenger RNA that encodes an amino acid. Synonymous codons encode the same amino acid, but species often prefer specific codons, leading to codon usage bias (CUB) (Grantham et al., 1980). Codon bias, influenced by mutation pressure, natural selection, and genetic drift (Bulmer, 1991), often correlates with higher gene expression (Ikemura, 1985; Sharp and Li, 1987). Mutation pressure causes deviations in nucleotide composition, while natural selection restricts codon usage bias to optimise protein production efficiency in highly expressed genes (Guan et al., 2018). Low-frequency codons support regulatory diversity of gene expression, playing an important role in the evolution and stability of species (Liu et al., 2010). Other influencing factors include nucleotide composition (Sueoka, 1962; Sueoka, 1988)), gene length (Eyre-Walker, 1996), expression level (Akashi, 1994), and tRNA abundance (Ikemura, 1981; 1985). In plants, monocotyledons prefer G/C-ending codons, while dicotyledons favor A/T-ending ones (Murray et al., 1989). Codon usage patterns help reveal evolutionary relationships among species (Dahal and bansal, 2025). GC content also serves as a distinguishing factor between these two groups of plants. Related species often exhibit similar codon usage biases, making it an important tool for inferring evolutionary relationships among different plant species (Morton, 1998; Muse and Gaut, 1994).

CUB reflects the origins, evolution, and mutations of species or genes, providing essential insights for gene function analysis, protein expression and protein structure research (Wu et al., 2015; He et al., 2016a). In recent years, studies on codon preference in gene families and individual genes across plants (Liu et al., 2020), animals (Uddin et al., 2020), viruses (Deb et al., 2020; Xin et al., 2020) and other organisms has been increasingly wide spread. WRKYs are among the largest plant transcription factor families, playing key roles in hormone synthesis, signaling, growth, metabolism and responses to biotic and abiotic stresses (Govardhana and Satyan, 2020; Li et al., 2020). The advent of next-generation sequencing technologies and bioinformatics tools has significantly enhanced comprehensive studies of *WRKY* transcription factors and their roles in plant adaptation to environmental stress (Wani et al., 2021; Goyal et al., 2023; Liu et al., 2023; Deng et al., 2024).

Banana (*Musa* spp.) is an important fruit crop, which supplies essential nutrients to millions of people around the world (Drenth and Kema, 2021). Cultivated Banana plants originated from natural intra- and inter specific hybridization between two diploid species, *Musa acuminata (*AA genome) and *Musa balbisiana* (BB genome) (Drenth and Kema, 2021). However, due to various biotic and abiotic stresses, global banana yields have significantly declined in recent years (Siamak and Zheng, 2018; Patel et al., 2019). One of the major diseases threatening the banana production dominated by ‘Cavendish’ from the AAA subgroup is the Fusarium wilt of banana, caused by *Fusarium oxysporum* f. sp. *cubense* tropical race 4 (*Foc*TR4) (Zorrilla-Fontanesi et al., 2020; Roberts et al., 2024). Current efforts have been focused on containment and deterrence through biosecurity measures, biological control agents, integrated disease management strategies, and the development of resistant banana varieties (Swarupa et al., 2014; Ploetz and Randy, 2015; Dita et al., 2018; Pegg et al., 2019). Resistant (R) genes, such as Resistance Gene Analog 2 (RGA2), which was isolated from *Foc*TR4-resistant *M*. *acuminata* ssp. *malaccensis*, demonstrated strong resistance in the field when over-expressed in ‘Cavendish’ transgenic lines (Dale et al., 2017). Forward genetic studies have also identified QTLs conferring resistance to *Foc*TR4 and subtropical race 4 (Ahmad et al., 2020; Chen et al., 2023a; 2023b). Candidate R genes, specifically pattern recognition receptors, were identified.

Several *WRKY* genes, such as *MaWRKY28, MaWRKY71, MaWRKY40 and MaWRKY22,* have been associated with banana’s response to biotic stress, including *Foc*TR4 (Sun et al., 2019; Li et al., 2012). However, research on the codon usage pattern of *WRKY* genes in banana has not been reported. Our previous research demonstrated that ‘Guijiao 9' (*Musa acuminata*) possesses strong resistance to *Foc*TR4, with lower disease incidence and severity compared to susceptible banana cultivars (Sun et al., 2019). We hypothesized that codon optimization in defense-related genes accelerates stress-responsive translation. Recent studies demonstrate that resistant plant varieties exhibit stronger codon bias in immune genes than susceptible counterparts (Dahal and bansal, 2025). We thus propose that CUB patterns in *WRKY* transcription factors, key regulators of Fusarium defense pathways (Javed and Gao, 2023), reflect evolutionary adaptations that facilitate rapid pathogen response in ‘Guijiao 9'. In this study, 151 members of *WRKY* gene family were identified from the ‘Guijiao 9′ transcriptome data. Factors influencing codon preference of *MaWRKY* and potential evolutionary models were determined by analyzing the CUB of *MaWRKY* genes in ‘Guijiao 9'. These findings contribute to understanding the function of the *WRKY* gene family and provide insights for codon optimisation of *MaWRKY* genes in the regulation of biotic stress in banana.

## 2 Methods

### 2.1 Screening, identification and characterization of sequence

The coding sequences of *WRKY* transcription factors were initially extracted from the transcriptome database of *Musa acuminata* cultivar ‘Guijiao 9' (Supplementary File 1). To ensure comprehensive identification of all *WRKY* family members, we implemented a rigorous multi-step screening protocol. First, local BLAST searches (e-value cutoff: 1e-5) were performed to obtain corresponding amino acid and nucleotide sequences. Candidate sequences were then subjected to domain verification using HMMER 3.3 with the WRKY domain profile (PF03106) from PFAM database as reference (E-value < 0.01). All potential *WRKY* sequences were further validated through NCBI’s Conserved Domain Database (CDD) and only those containing the complete WRKYGQK motif were retained. Finally, open reading frames were predicted using ORFfinder with a minimum length requirement of 300 bp to ensure sequence integrity. Through this stringent screening pipeline, we identified 151 high-confidence *MaWRKY* genes with complete WRKY domains and intact coding sequences, which were subsequently used for comprehensive codon usage analysis (Supplementary File 2).

### 2.2 Indices of codon usage bias

Following the screening of *MaWRKY* gene CDS sequences, the key indices of CUB were calculated, including the effective number of codons (ENC), relative synonymous codon usage (RSCU), total GC content of each CDS (GC), and GC content at the first, second and third codon positions (GC1, GC2, GC3). Additionally, the nucleotide composition at synonymous third codon positions (A3s, T3s, G3s, C3s) was analyzed. These calculations were performed using the CodonW program (version 1.4.2) (Peden, 1986), with parameters validated for monocot genomes (Wright, 1990). Correlations between nucleotide contents were assessed using SPSS statistical software (version 23.0), with values exceeding 0.5 and below-0.5 indicating strong positive and strong negative correlations, respectively.

ENC is a standard indicator of CUB, with values from 20 (complete bias, one codon per amino acid) to 61 (no bias, equal usage of all synonymous codons) (Lu et al., 2005), which reflects the degree of CUB (Gao et al., 2022). The CUB is considered low if the ENC value is greater than 40 (Wright, 1990). RSCU measures the observed frequency of a codon relative to its frequency expected at random, independent of the amino acid composition of gene product (Wang et al., 2016). RSCU values equal to one imply uniform codon usage, values greater than one suggest preferential usage (positive bias), and values less than one denote reduced usage (negative bias) (Sharp and Li, 1986).

### 2.3 ENC-plot analysis

ENC-plot analysis is commonly used to identify factors influencing CUB by plotting ENC values against GC3s values. The standard curve represents the relationship between ENC and GC3s, helping to distinguish whether mutation pressure or natural selection is the dominant factor (Wright, 1990). When CUB is primarily driven by mutation pressure, data points typically lie on or just below the standard curve. In contrast, if natural selection and other factors play a larger role, the points tend to fall noticeably below the standard curve (Wright, 1990). However, the ENC-GC3s plot alone cannot conclusively distinguish the dominant force, because strong mutation pressure may push points below the curve, mimicking selection, and weak selection signals may be masked by mutational noise (Fuglsang, 2004; Plotkin and Kudla, 2011). Therefore, we employed complementary approaches including Parity Rule 2 Bias analysis, Neutrality Plot and Principle Component Analysis on RSCU to resolve this ambiguity.

### 2.4 Parity Rule 2 (PR2) bias plot analysis

A PR2 bias plot is created by plotting the AT bias (A3/(A3+ T3)) on the y-axis and the GC bias (G3/(G3 + C3)) on the x-axis. The midpoint at 0.5 indicates an equal balance between G = C and A = T, suggesting no significant bias from either mutation or selection pressure (Deb et al., 2020). If the genes are clustered near the center, it suggests that the base frequencies are relatively balanced, and the CUB is mainly affected by mutation pressure. Conversely, if the genes are far from the center, other factors may be affecting the CUB.

### 2.5 Neutrality plot analysis

A neutrality plot is used to assess the extent to which CUB is influenced by mutation *versus* selection in organisms, by comparing GC3 (x-axis) and GC12 (y-axis). In this plot, each gene is represented by a point. If the regression coefficient of the plot approaches 1 (complete neutrality), the points will show a clear pattern, indicating selection plays a significant role in CUB. If the regression coefficient deviates from 1, it suggests that factors other than mutation pressure are also influencing the CUB (Sueoka, 1988).

### 2.6 Principal Component Analysis (PCA) on RSCU

Principal Component Analysis or PCA is a multivariate statistical method to examine relationships among multiple variables, and is often applied to analyze trends in synonymous codon usage patterns (Kanaya et al., 1996). In this study, the RSCU values for 56 synonymous codons from *MaWRKY* genes was reduced from 151 dimensions (representing 151 *MaWRKY* genes) to two principal components through dimensionality reduction. Fifty-six synonymous codons did not include the initiation codon AUG, tryptophan codon UGG, the three triplet codons for Isoleucine, and the termination codons UAA, UAG, and UGA (Sueoka, 1988; Zhang et al., 2018).

### 2.7 Optimal codon analysis

The optimal codon plays a significant role in improving the speed and accuracy of translation (Duret and andMouchiroud, 1999). To predict the optimal codon for *MaWRKY*, the ΔRSCU method was applied (Sharp and Li, 1987; Ikemura, 1985). The 151 *MaWRKY* gene sequences were sorted based on their ENC values. The top 10% (n = 15) with the lowest ENC values and the bottom 10% (n = 15) with the highest ENC values were selected as extreme groups for optimal codon identification, and the RSCU values for the codons were calculated. A codon was designated as a high-expression superior codon if its RSCU value was higher in the high-expression group than in the low-expression group, and the difference between groups (ΔRSCU) met the threshold of ΔRSCU ≥ 0.08. These candidate codons were subsequently evaluated against the complete dataset. A codon was classified as an optimal codon only if its genome-wide RSCU value exceeded 1.0.

### 2.8 Statistical analysis

The CDS sequences of the *MaWRKY* were analyzed using CodonW (http://codonw.sourceforge.net/) and EMBOSS online software (http://www.bioinformatics.nl/emboss-explorer/). Codon-related parameters were statistically analyzed using Microsoft Office Excel 2016. ENC-plot analysis, Neutrality plot, and Box diagram were generated using Origin8.0 and HemI 1.0 (http://hemi.biocuckoo.org/down.php) software.

## 3 Results

### 3.1 Codon composition of *MaWRKY* genes reveals G/C bias

**FIGURE 1:**
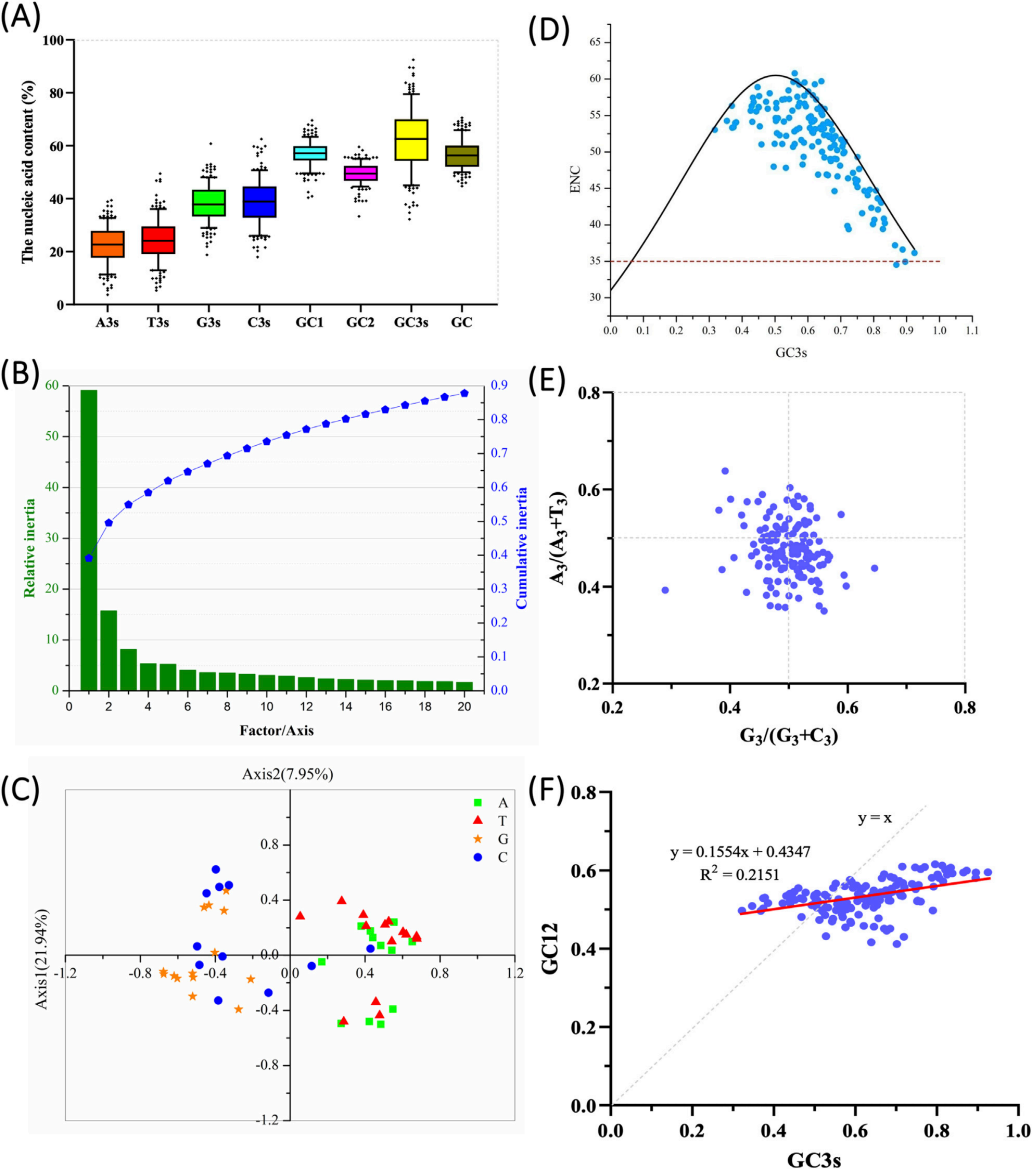
Codon usage analysis of the *WRKY* gene family in *Musa acuminata* ‘Guijiao 9’. **(A)** Nucleic acid composition of *MaWRKY* codons. The Y-axis represents the nucleic acid content, while the X-axis corresponds to the total GC content of each coding domain sequence (CDS), the nucleotide composition at the third codon position (A3s, T3s, G3s, C3s),the GC content at each codon position of GC1, GC2, and GC3 (representing the first, second, and third positions, respectively) for each CDS. **(B)** Principal component analysis (PCA) on the relative and cumulative contributions of the first 20 factors to the total variance. **(C)** PCA plot showing codon RSCU values on codons ending with A, T, G, or C. Green squares represent codons ending with A; red triangles indicate those ending with T, orange stars indicate codons ending with G, and blue circles indicate codons ending with C. **(D)** ENC-plot analysis of *MaWRKY* against GC3s. Points on or near the curve indicate bias caused by mutation pressure, while points away from the curve suggest influence from natural selection or other factors. The red-dotted line means the lowest ENC value of *MaWRKY.* **(E)** PR2 plot analysis. The midpoint at 0.5 represents an equal balance between G = C and A = T, indicating no bias between mutation and selection pressure. Deviations from 0.5 suggest that codon bias is primarily influenced by factors other than base mutation of gene-encoding amino acids. **(F)** Neutrality plot analysis of *MaWRKY*. The plot compares GC3 (x-axis) and GC12 (y-axis). A regression coefficient value less than 1.0 (complete neutrality) indicates an influence of natural selection or mutation pressure. The red straight line represents the fitted curve.

The codon composition and ENC values of 151 banana *MaWRKY* family members were statistically analyzed (Figure 1), with detailed codon bias parameters provided in Supplementary File 2. The ENC values ranged from 35.03 to 60.14, indicating varying levels of codon bias, although the overall CUB was weak. The average content of A3s and T3s was not significantly different from G3s and C3s (p > 0.05) (Figure 1A). However, the combined average content of G3s + C3s differed significantly from that of A3s + T3s. The average GC content was 56.55%, ranging from 44.7% to 70.6%, indicating a relatively high GC content and a preference for G/C-ending codons in *MaWRKY* genes. Analysis of G/C content at different codon positions showed that GC3s (62.23% on average) were much higher than GC1s and GC2s, suggesting preference for G or C at the third codon position. The first and second codon positions are relatively stable, while the third position shows greater fluctuation.

### 3.2 Codon usage parameter correlation analysis

The Pearson Correlation Coefficient for various codon usage indices was calculated and their values are presented in Table 1 to assess the relationships between factors influencing codon usage. ENC value showed a significant positive correlation with A3s (r = 0.706, p < 0.05) and T3s (r = 0.690, p < 0.05) but was negatively correlated with G3s (r = −0.609, p < 0.05), C3s (r = −0.691, p < 0.05), GC (r = −0.687, p < 0.05) and GC3s (r = −0.737, p < 0.05).These findings indicate that the nucleotide composition at the third position of synonymous codons has some subtle influence on CUB.

**TABLE 1:** Pearson correlation coefficients among parameters influencing codon bias in *MaWRKY* genes of *Musa acuminata* banana ‘Guijiao 9'. A3s, T3s, G3s and C3s denote the nucleotide content at the third codon position. GC represents the overall GC content of each coding domain sequence, while GC1, GC2, and GC3 refer to the GC content at the first, second, and third codon positions, respectively. ENC indicates Effective Number of Codons.

Indices	A3s	T3s	G3s	C3s	GC	GC1	GC2	GC3s	ENC
A3s	1.000								
T3s	0.811^**^	1.000							
G3s	−0.806^**^	−0.774^*^	1.000						
C3s	−0.841^**^	−0.908^**^	0.610^*^	1.000					
GC	−0.897^**^	−0.877^**^	0.679^*^	0.842^**^	1.000				
GC1s	−0.481	−0.373	0.228	0.370	0.706^*^	1.000			
GC2s	−0.432	−0.428	0.111	0.370	0.673^*^	0.546	1.000		
GC3s	−0.941^**^	−0.957^**^	0.844^**^	0.927^**^	0.912^**^	0.415	0.400	1.000	
ENC	0.706^*^	0.690^*^	−0.609^*^	−0.691^*^	−0.687^*^	−0.363	−0.286	−0.737^*^	1.000

* and ** indicate *p* values < 0.05 and < 0.01, respectively.

Additionally, G3s was positively correlated with C3s (r = 0.610, p < 0.05), GC3s (r = 0.844, p < 0.01) and GC (r = 0.679, p < 0.05). Conversely, A3s exhibited a negative correlation with G3s (r = −0.806, p < 0.01), C3s (r = −0.841, p < 0.01), GC3s (r = −0.941, p < 0.01) and GC (r = −0.897, p < 0.01). Similarly, T3s showed a negative correlation with G3s (r = −0.774, p < 0.05), C3s (r = −0.908, p < 0.01), GC3s (r = −0.957, p < 0.01), and GC (r = −0.877, p < 0.01) (Table 1). The lack of significant positive correlations indicates that A/T-ending codon usage frequency is not a primary determinant of CUB variation in *MaWRKY* genes, though minor influences cannot be excluded.

### 3.3 Principal Component Analysis

Principal Component Analysis (PCA) was performed on the RSCU values of *MaWRKY* genes sequences. The contribution of the first 20 factors to the variance in CUB is shown in Figure 1B. The first four factors accounted for 58.47% of the total variance, capturing the majority of the differences in codon usage patterns. Axis1, which accounted for the largest proportion of variance, was the principal axis explaining CUB and reflected the major source of variation in codon usage. PCA analysis of the 56 codons in *MaWRKY* family members, categorized by their endings (A, T, C or G), revealed distinct distribution patterns (Figure 1C). A/T codons formed dense clusters, contrasting with broadly distributed G/C codons, with those ending in C being spread across all four quadrants. These findings indicate a natural evolutionary or long-time domestication bias in banana codon usage toward G and C endings.

### 3.4 Factors affecting CUB in *MaWRKY* genes of *Musa acuminata*

ENC-plot analysis is commonly used to assess the effect of mutation pressure on CUB. The ENC values are plotted against GC3 values (Figure 1D). The standard curve shows that the relationship between ENC and GC3s is shaped primarily by mutation pressure rather than selection. The ENC values for *MaWRKY* family members generally align with the standard curve in the ENC-plot, indicating that mutation pressure is a major factor influencing CUB for these genes (Figure 1D). However, the ENC values of many *MaWRKY* genes deviate significantly from the standard curve (Figure 1D), suggesting that mutation pressure is not the sole factor driving codon bias. Other factors, such as natural selection and gene expression, may also contribute to the observed codon bias.

PR2 analysis was performed to assess the impact of mutation and selection pressure on codon usage by examining whether there was a mutation imbalance between A/T (U) and C/G. In the PR2 plot, A3/(A3 + T3) is plotted as the ordinate and G3/(G3+C3) as the abscissa for *MaWRKY* family members to explore the influence of evolutionary factors (Figure 1E). It could be seen that codon T and C of *MaWRKY* in the third position has a slight bias. It was in line with the view of that C and T are used more frequently than G and A in four-fold degenerate codon groups in monocot and dicot plant species (Kawabe and Miyashita, 2003). The A3/(A3 + T3) or G3/(G3 + C3) values for most *MaWRKY* genes deviated from 0.5, suggesting that codon bias was additionally influenced by factors other than base mutation in codon sequences (Figure 1E). This indicates that additional pressures, such as natural selection, likely played a role in the evolution of *MaWRKY* genes.

Neutrality plot analysis was performed by comparing GC3 (abscissa) and GC12 (ordinate) to examine the role of mutation-selection equilibrium in codon usage variation. A linear regression line was plotted with GC3 (abscissa) and GC12 (ordinate) values (Figure 1F). When the slope of the regression line approaches 1, it suggests that mutation pressure plays a dominant role in shaping CUB, whereas a slope near 0 indicates that natural selection is the primary influencing factor (Sueoka, 1988). In Figure 1F, the low coefficient of determination (*R* ^2^ = 0.2151) indicates that only 21.5% of the variation in GC12 can be explained by GC3, suggesting that mutational pressure plays a limited role in shaping codon usage patterns. Moreover, the low slope value (0.1554) implies that the GC content at the third codon position has minimal influence on the first and second positions, further supporting the hypothesis that natural selection is the dominant force influencing CUB in *MaWRKY* genes (Figure 1F).

### 3.5 Relative synonymous codon usage (RSCU) in *MaWRKY*gene family

**FIGURE 2:**
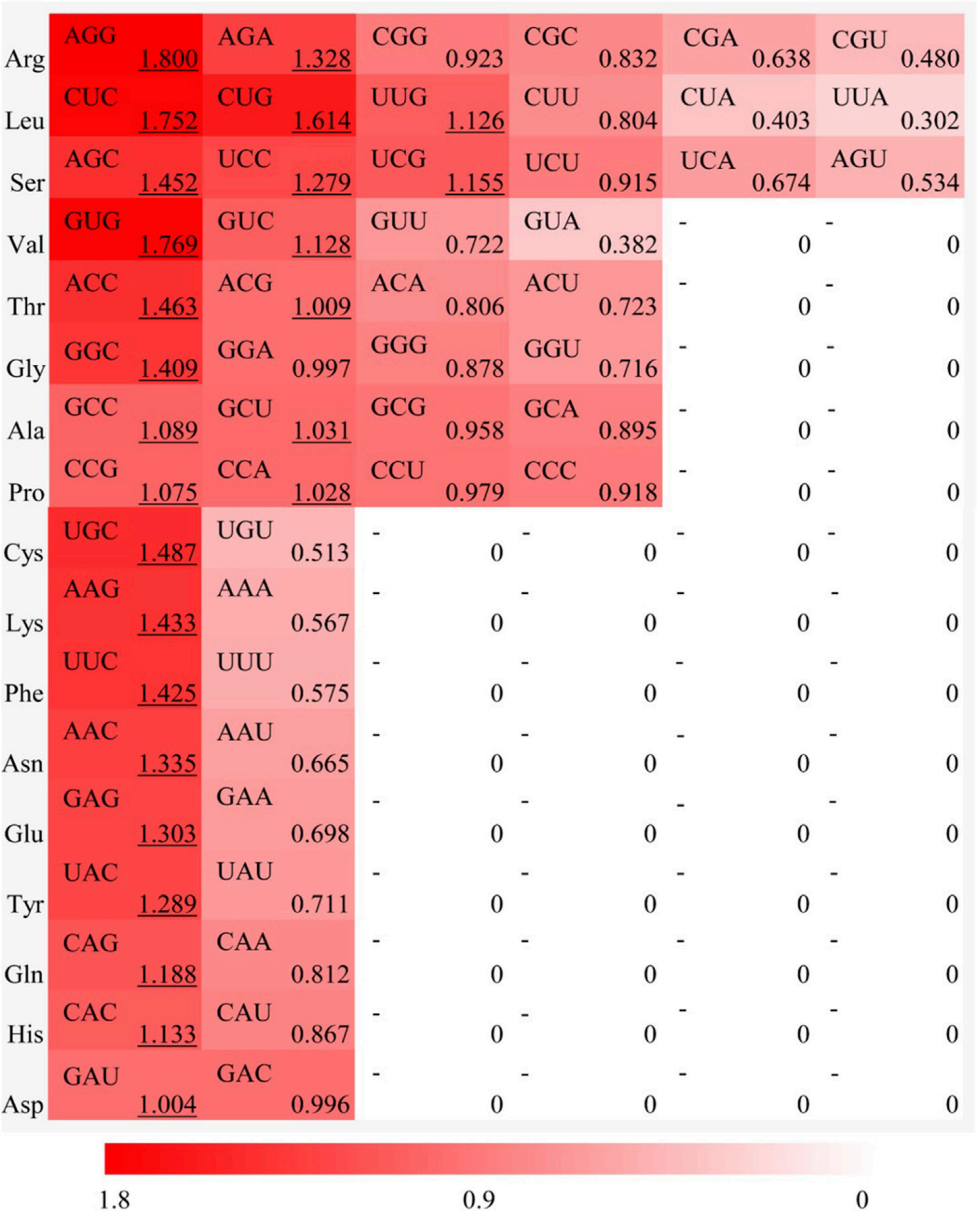
Relative synonymous codon usage (RSCU) analysis for each amino acid in Ma*WRKY* gene family of *Musa acuminata* banana ‘Guijiao 9'. The red color bar represents the RSCU value, with deeper shades indicating higher RSCU values.

To investigate the patterns of synonymous codon usage bias in *MaWRKY* gene family, the RSCU of 56 synonymous codons (excluding the initiation and termination, isoleucine, and tryptophan codons) were analyzed. The results revealed 26 high-frequency codons (RSCU > 1) in *MaWRKY* family members, including AGG, AGA, CUC, CUG, UUG, AGC, UCC, UCG, GUG, GUC, ACC, ACG, GGC, GCC, GCU, CCG, CCA, UGC, AAG, UUC, AAC, GAG, UAC, CAG, CAC, and GAU, respectively (Figure 2). Among these, 22 codons ended in G/C, indicating a preference for G or C ending codons in *MaWRKY* members. The codon AGG of Arginine (RSCU = 1.800) exhibited the strongest preference, accounting for 30% of the synonymous codons. These high-frequency codons show preferential usage, which contributed to the deviation of *MaWRKY* genes from an ENC value of 61.

### 3.6 The optimal codons of *MaWRKY* genes

The optimal codons in *MaWRKY* genes of *M*. *acuminata* ‘Guijiao 9′ were identified by combining high-frequency codons (RSCU > 1) and highly expressed codons (ΔRSCU ≥ 0.08). The analysis revealed 24 highly expressed codons with ΔRSCU ≥ 0.08 (Table 2) and 26 high-frequency codons with RSCU > 1 (Figure 2). In total, 15 optimal codons were determined for *MaWRKY* genes in *M*. *acuminata* ‘Guijiao 9′, including CUC, CUG, GUG, UCC, AGC, CCG, ACC, GCC, UAC, CAC, CAG, AAC, GAG, UUC and GGC. Notably, all of these optimal codons end with G or C (Table 2).

**TABLE 2:** Identification of optimal codons in *MaWRKY* genes of *M. acuminata* ‘Guijiao 9'.

AA	Codon	RSCU		ΔRSCU	AA	Codon	RSCU		ΔRSCU
		High expression	Low expression				High expression	Low expression	
Leu (L)	UUA	0.37	0.4	−0.03	Tyr (Y)	UAU	0.48	0.73	−0.26
	UUG	0.48	1.63	−1.14		UAC ^*^	1.52	1.27	0.26
	CUU^*^	0.73	0.69	0.04	His (H)	CAU	0.77	1.27	−0.5
	CUC ^*^	2.13	1.1	1.03		CAC ^*^	1.23	0.73	0.5
	CUA	0.37	0.53	−0.16	Gln (Q)	CAA	0.69	1.21	−0.52
	CUG ^*^	1.92	1.66	0.26		CAG ^*^	1.31	0.79	0.52
Val (V)	GUU	0.56	0.69	−0.14	Asn (N)	AAU	0.4	0.99	−0.59
	GUC	1	1.24	−0.24		AAC ^*^	1.6	1.02	0.59
	GUA	0.29	0.47	−0.18	Lys (K)	AAA	0.49	0.5	−0.01
	GUG ^*^	2.16	1.59	0.57		AAG	1.51	1.5	0.01
Ser (S)	UCU	0.67	0.89	−0.22	Asp (D)	GAU	0.79	1.23	−0.44
	UCC ^*^	1.43	0.64	0.8		GAC^*^	1.21	0.77	0.44
	UCA	0.37	0.95	−0.58	Glu (E)	GAA	0.4	0.78	−0.38
	AGU^*^	0.27	0.4	−0.13		GAG ^*^	1.6	1.22	0.38
	AGC ^*^	2.12	1.9	0.22	Cys (C)	UGU	0.41	0.61	−0.2
	UCG	1.14	1.22	−0.08		UGC	1.44	1.39	0.05
Pro (P)	CCU	1.07	1.36	−0.29	Phe (F)	UUU	0.31	0.68	−0.37
	CCC	1.02	0.7	0.32		UUC ^*^	1.69	1.32	0.37
	CCA	0.6	1.34	−0.74	Arg (R)	CGU^*^	0.71	0.12	0.59
	CCG ^*^	1.31	0.6	0.71		CGC^*^	1.29	0.3	0.98
Thr (T)	ACU	0.74	0.81	−0.08		CGA^*^	0.49	0.41	0.08
	ACC ^*^	1.69	0.51	1.18		CGG^*^	1.11	0.68	0.42
	ACA	0.43	1.53	−1.1		AGA	1.23	2.12	−0.9
	ACG	1.14	1.15	−0.01		AGG	1.19	2.36	−1.18
Ala (A)	GCU	1.05	1.12	−0.07	Gly (G)	GGU^*^	0.72	0.38	0.34
	GCC ^*^	0.99	0.62	0.37		GGC ^*^	2.05	1.63	0.42
	GCA^*^	1	0.89	0.12		GGA	0.75	1.08	−0.33
	GCG	0.96	1.38	−0.42		GGG	0.63	0.9	−0.28

* indicates highly expressed codons (ΔRSCU ≥ 0.08), the underlined text represents high-frequency codons (RSCU > 1), and the bold text highlights the optimal codon in *MaWRKY*, genes of *M. acuminata* ‘Guijiao 9'.

## 4 Discussion

During the process of gene expression, the selective use of codons varies from species to species. Different organisms exhibit different preference for synonymous codons encoding the same amino acid, closely linked to their genetic characteristics. CUB can influence mRNA stability, transcription, protein translation accuracy, and protein folding, thereby fine-tuning gene expression. In the present study, 151 coding sequences of *WRKY* genes from *M. acuminata* ‘Guijiao 9′ were analyzed to investigate CUB and its potential influencing factors. The results revealed that *MaWRKY* genes in *M*. *acuminata* ‘Guijiao 9′ exhibit a preference for codons ending in G/C, similar to other monocotyledons, such as *Musa basjoo*, *Zea mays* and *oryza sativa* (Ma et al., 2015).

Overall, the CUB was weak, and the expression levels of these genes were low, indicating high variability in synonymous codon usage among *MaWRKY* genes. This is not surprising since the expression of *WRKY* genes is known to be induced by stress (Dong et al., 2003; Kayum et al., 2015). *WRKY* transcription factors exhibit constitutively low expression in non-stress environments through evolutionarily optimized transcriptional restraint (Rushton et al., 2010; Birchler and Yang, 2022), preventing unnecessary resource allocation (Tian et al., 2003) while enabling rapid stress response mobilization via chromatin poising (Li et al., 2020). Furthermore, the GC3 content showed greater variability than GC1 and GC2 with codons ending in G/C exhibiting a broad distribution. These results suggest that nucleotide composition plays a significant role in influencing CUB in *MaWRKY* genes. While mutation pressure played a role, natural selection was likely the primary factor in shaping the codon usage patterns of *MaWRKY* genes during evolution. In *Ginkgo biloba*, it was reported that certain genes were involved in environment adaptation preferentially using G/C-ending codons, with natural selection as the primary driver of CUB (He et al., 2016b). Interestingly, while monocotyledons like *M. acuminata* favor G/C-ending codons, dicotyledons such as *Helianthus annuus* (Gao et al., 2022) and *Brassica napus* (Li et al., 2013) exhibit a preference for A/T (U)-ending codons. In these dicotyledonous plant species, mutation pressure appears to play a larger role in shaping CUB (Gao et al., 2022; Li et al., 2013). Thus, the codon usage preferences vary significantly among different plant species, reflecting distinct evolutionary pressures and adaptations.

This work establishes codon usage patterns as evolutionary signatures of historical selection pressures, creating a predictive framework for future functional studies on WRKY-mediated stress responses in banana.

## Data Availability

The original contributions presented in the study are included in the article/Supplementary Material, further inquiries can be directed to the corresponding author. Publicly available datasets were analyzed in this study. This data can be found in NCBI, https://www.ncbi.nlm.nih.gov/datasets/taxonomy/214687/

## Abbreviations

CUB, Codon usage bias; ENC, Effective number of codons; RSCU, Relative synonymous codon usage; PCA, Principal component analysis; PR2, Parity Rule 2; GC1, GC2, GC3, The GC content at the first, second and third position, respectively; A3s, T3s, G3s, C3s, The content of each nucleotide of the codon at synonymous third positions.

## References

[B1] Ahmad F. Martawi N. M. Poerba Y. S. Hans S. Henk K. Gert H. J. (2020). Genetic mapping of fusarium wilt resistance in a wild banana *musa Acuminata*ssp. *Malaccensis* accession. Theor. Appl. Genet. 133 (12), 3409–3418. 10.1007/s00122-020-03677-y 32918589 PMC7567712

[B2] Akashi H. (1994). Synonymous codon usage in *drosophila Melanogaster*: natural selection and translational accuracy. Genetics 136 (3), 927–935. 10.1093/genetics/136.3.927 8005445 PMC1205897

[B3] Birchler J. A. Yang H. (2022). The multiple fates of gene duplications: deletion, hypofunctionalization, subfunctionalization, neofunctionalization, dosage balance constraints, and neutral variation. Plant Cell. 34 (7), 2466–2474. 10.1093/plcell/koac076 35253876 PMC9252495

[B4] Bulmer M. (1991). The selection-mutation-drift theory of synonymous codon usage. Genetics 129 (3), 897–907. 10.1093/genetics/129.3.897 1752426 PMC1204756

[B7] Chen A. Sun J. M. Martin G. Gray L. A. Hribová E. Christelová P. (2023a). Identification of a major QTL-Controlling resistance to the subtropical race 4 of *fusarium Oxysporum*f. Sp. *cubense*in *Musa acuminata* ssp. *Malaccensis* . Pathogens 12 (2), 289. 10.3390/pathogens12020289 36839561 PMC9964652

[B8] Chen A. Sun J. M. Viljoen A. Mostert D. Xie Y. C. Mangila L. (2023b). Genetic mapping, candidate gene identification and marker validation for host plant resistance to the race 4 of *Fusarium oxysporum* F. sp. *cubense* using *Musa acuminata* ssp. *malaccensis* . Pathogens 12 (6), 820. 10.3390/pathogens12060820 37375510 PMC10303076

[B9] Dahal U. bansal A. (2025). Codon usage and antibiotic resistance: a hidden evolutionary mechanism. Biochimie. 10.1016/j.biochi.2025.07.027 40752602

[B10] Dale J. James A. Paul J. Y. Khanna H. Smith M. Peraza-Echeverria S. (2017). Transgenic Cavendish bananas with resistance to Fusarium wilt tropical race 4. Nat. Commun. 8 (1), 1496. 10.1038/s41467-017-01670-6 29133817 PMC5684404

[B11] Deb B. Uddin A. Chakraborty S. (2020). Codon usage pattern and its influencing factors in different genomes of hepadnaviruses. Arch. Virol. 165, 557–570. 10.1007/s00705-020-04533-6 32036428 PMC7086886

[B13] Deng P. J. Wang Z. Li W. Y. Chen X. H. Liu D. Q. (2024). WRKY11 up-regulated dirigent expression to enhance lignin/lignans accumulation in Lilium regale Wilson during response to Fusarium wilt. J. Inte Agri. 23 (8), 2703–2722. 10.1016/j.jia.2023.07.032

[B14] Dita M. Barquero M. Heck D. Mizubuti E. S. G. Staver C. P. (2018). Fusarium wilt of banana: current knowledge on epidemiology and research needs toward sustainable disease management. Front. Plant Sci. 9, 1468. 10.3389/fpls.2018.01468 30405651 PMC6202804

[B15] Dong J. Chen C. Chen Z. (2003). Expression profiles of the Arabidopsis WRKY gene superfamily during plant defense response. Plant Mol. Biol. 51, 21–37. 10.1023/a:1020780022549 12602888

[B16] Drenth A. Kema G. (2021). The vulnerability of bananas to globally emerging disease threats. Phytopathology 111, 2146–2161. 10.1094/PHYTO-07-20-0311-RVW 34231377

[B18] Duret L. Mouchiroud D. (1999). Expression pattern and, surprisingly, gene length shape codon usage in Caenorhabditis, Drosophila, and Arabidopsis. PANS 96, 4482–4487. 10.1073/pnas.96.8.4482 10200288 PMC16358

[B19] Eyre-Walker A. (1996). Synonymous codon bias is related to gene length in *Escherichia coli*: selection for translational accuracy? Mol. Biol. Evol. 13 (6), 864–872. 10.1093/oxfordjournals.molbev.a025646 8754221

[B20] Fuglsang A. (2004). The 'effective number of codons' revisited. Biochem. Biophys. Res. Commun. 317 (3), 957–964. 10.1016/j.bbrc.2004.03.138 15081433

[B21] Gao Y. Lu Y. Song Y. Jing L. (2022). Analysis of codon usage bias of WRKY transcription factors in *Helianthus annuus* . BMC Genomic Data 23, 46. 10.1186/s12863-022-01064-8 35725374 PMC9210703

[B22] Govardhana M. Satyan K. B. (2020). *In-silico* Analysis of cucumber (*Cucumis sativus* L.) genome for WRKY transcription factors and cis-acting elements. Comput. Bio Chem. 85, 107212. 32058944 10.1016/j.compbiolchem.2020.107212

[B23] Goyal P. Devi R. Verma B. Hussain S. Arora P. Tabassum R. (2023). WRKY transcription factors: evolution, regulation, and functional diversity in plants. Protoplasma. 260, 331–348. 10.1007/s00709-022-01794-7 35829836

[B24] Grantham R. Gautier C. Gouy M. Mercier R. Pavé A. (1980). Codon catalog usage and the genome hypothesis. Nucleic Acids Res. 8 (1), 49–62. 10.1093/nar/8.1.197-c 6986610 PMC327256

[B25] Guan D. L. Ma L. B. Salabat K. M. Zhan X. X. Xu S. Q. Xie J. Y. (2018). Analysis of codon usage patterns in *Hirudinariamanillensis* reveals a preference for GC-ending codons caused by dominant selection constraints. BMC Genomics 19, 542. 10.1186/s12864-018-4937-x 30016953 PMC6050667

[B26] He B. Dong H. Jiang C. Cao F. Xu L. A. (2016a). Analysis of codon usage patterns in *Ginkgo biloba* reveals codon usage tendency from A/U-ending to G/C-ending. Sci. Rep. 6, 35927. 10.1038/sre35927 27808241 PMC5093902

[B27] He B. Dong H. Jiang C. Cao F. L. Tao S. T. Xu L. A. (2016b). Analysis of codon usage patterns in *Ginkgo biloba*reveals codon usage tendency from A/U-ending to G/C ending. Sci. Rep. 6, 35927. 10.1038/srep35927 27808241 PMC5093902

[B28] Ikemura T. (1985). Codon usage and tRNA content in unicellular and multicellular organisms. Mol. Biol. Evol. 2 (1), 13–34. 10.1093/oxfordjournals.molbev.a040335 3916708

[B70] Ikemura T. (1981). Correlation between the abundance of Escherichia coli transfer RNAs and the occurrence of the respective codons in its protein genes: a proposal for a synonymous codon choice that is optimal for the E. coli translational system. J. Mol. Biol. 151, 389–409. 6175758 10.1016/0022-2836(81)90003-6

[B29] Javed T. Gao S. (2023). WRKY transcription factors in plant defense. Trends Genet. 39 (10), 787–801. 10.1016/j.tig.2023.07.001 37633768

[B30] Kanaya S. Kudo Y. Nakamura Y. Ikemura T. (1996). Detection of genes in *Escherichia coli* sequences determined by genome projects and prediction of protein production levels, based on multivariate diversity in codon usage. Bioinformatics 12, 213–225. 10.1093/bioinformatics/12.3.213 8872390

[B31] Kawabe A. Miyashita N. T. (2003). Patterns of codon usage bias in three dicot and four monocot plant species. Genes and Genet. Syst. 78 (5), 343–352. 10.1266/ggs.78.343 14676425

[B32] Kayum M. A. Jung H. J. Park J. I. Ahmed N. U. Saha G. Yang T. J. (2015). Identification and expression analysis of WRKY family genes under biotic and abiotic stresses in *Brassica rapa* . Mol. Genet. Genomics 290, 79–95. 10.1007/s00438-014-0898-1 25149146

[B34] Li C. Y. Deng G. M. Yang J. Viljoen A. Jin Y. Kuang R. B. (2012). Transcriptome profiling of resistant and susceptible Cavendish banana roots following inoculation with *Fusarium oxysporum* f. sp*cubense*tropical race 4. BMC Genom 13, 374. 10.1186/1471-2164-13-374 22863187 PMC3473311

[B35] Li G. Y. Wang Z. Zhang Z. Y. Fang H. D. Tan X. L. (2013). The base composition and codon use of the WRKY gene family of the *Brassica napus* . J. Biol. 30, 42–45.

[B36] Li W. Zhu Z. Chern M. Yin J. Yang C. Wang J. (2017). A natural allele of a transcription factor in rice confers broad-spectrum blast resistance. Cell. 170 (1), 114–126.e1. 10.1016/j.cell.2017.06.008 28666113

[B37] Liu H. He R. Zhang H. Huang Y. Tian M. Zhang J. (2010). Analysis of synonymous codon usage in *Zea mays* . Mol. Biol. Rep. 37, 677–684. 10.1007/s11033-009-9521-7 19330534

[B38] Liu H. Lu Y. Lan B. Xu J. (2020). Codon usage by chloroplast gene is bias in*Hemipteleadavidii* . J. Genet. 99, 8. 10.1007/s12041-019-1167-1 32089527

[B39] Liu J. N. Fang H. C. Liang Q. Dong Y. H. Wang C. X. Yan L. P. (2023). Genomic analyses provide insights into the evolution and salinity adaptation of halophyte *Tamarix chinensis* . GigaScience 12, giad053–17. 10.1093/gigascience/giad053 37494283 PMC10370455

[B40] Lu H. Zhao W.-M. Zheng Y. Hong W. Mei Q. Yu X.-P. (2005). Analysis of synonymous codon usage bias in Chlamydia. Acta Biochim. Biophys. Sin. (Shanghai) 37 (1), 1–10. 10.1093/abbs/37.1.1 15645075 PMC7110193

[B68] Ma Q. Li C. Wang J. Wang Y. Ding Z. (2015). Analysis of synonymous codon usage in FAD7 genes from different plant species. Genet. Mol. Res. 14, 1414–1422. 25730080 10.4238/2015.February.13.20

[B41] Morton B. R. (1998). Selection on the codon bias of chloroplast and cyanelle genes in different plant and algal lineages. J. Mol. Evol. 46 (4), 449–459. 10.1007/pl00006325 9541540

[B42] Murray E. E. Lotzer J. Eberle M. (1989). Codon usage in plant genes. Nucleic Acids Res. 17, 477–498. 10.1093/nar/17.2.477 2644621 PMC331598

[B67] Muse S. V. Gaut B. S. (1994). A likelihood approach for comparing synonymous and nonsynonymous nucleotide substitution rates, with application to the chloroplast genome. Molecular Biology and Evolution, 11 (5), 715–724. 10.1093/nar/17.2.477 7968485

[B43] Patel P. Yadav K. Srivastava A. K. Suprasanna P. Ganapathi T. R. (2019). Overexpression of native Musa-miR397 enhances plant biomass without compromising abiotic stress tolerance in banana. Sci. Rep. 9, 16434–15. 10.1038/s41598-019-52858-3 31712582 PMC6848093

[B44] Peden A. M. (1986). Codon usage in plants. UK: University of Nottingham. Ph.D. Thesis.

[B45] Pegg K. G. Coates L. M. O’Neill W. T. Turner D. W. (2019). The Epidemiology of Fusarium wilt of banana. Front. Plant Sci. 10, 1395. 10.3389/fpls.2019.01395 31921221 PMC6933004

[B46] Ploetz R. C. Randy C. (2015). Fusarium wilt of banana. Phytopathology 105 (12), 1512–1521. 10.1094/PHYTO-04-15-0101-RVW 26057187

[B47] Plotkin J. Kudla G. (2011). Synonymous but not the same: the causes and consequences of codon bias. Nat. Rev. Genet. 12, 32–42. 10.1038/nrg2899 21102527 PMC3074964

[B49] Roberts J. M. Carvalhais L. C. O'Dwyer C. Rincón-Flórez V. A. Drenth A. (2024). Diagnostics of Fusarium wilt in banana: current status and challenges. Plant Pathol. 73 (4), 760–776. 10.1111/ppa.13863

[B50] Rushton P. J. Somssich I. E. Ringler P. Shen Q. J. (2010). WRKY transcription factors. Trends Plant Sci, 15 (5), 247–258. 10.1016/j.tplants.2010.02.006 20304701

[B51] Sharp P. M. Li W. H. (1986). Codon usage in regulatory genes in *Escherichia coli* does not reflect selection for ‘rare’codons. Nucleic acids Res. 14, 7737–7749. 10.1093/nar/14.19.7737 3534792 PMC311793

[B52] Sharp P. M. Li W. H. (1987). The codon adaptation index-a measure of directional synonymous codon usage bias, and its potential applications. Nucleic Acids Res. 15, 1281–1295. 10.1093/nar/15.3.1281 3547335 PMC340524

[B54] Siamak S. B. Zheng S. (2018). Banana Fusarium wilt (*Fusarium oxysporum* f. sp. *cubense*) control and resistance, in the context of developing wilt-resistant bananas within sustainable production systems. Horti Plant J. 4, 208–218. 10.1016/j.hpj.2018.08.001

[B55] Sueoka N. (1988). Directional mutation pressure and neutral molecular evolution. PANS 85, 2653–2657. 10.1073/pnas.85.8.2653 3357886 PMC280056

[B69] Sueoka N. (1988). On the genetic basis of variation and heterogeneity of DNA base composition. Proc. Natl. Acad. Sci. USA. 48, 582–592. 10.1073/pnas.85.8.2653 13918161 PMC220819

[B56] Sun J. Zhang J. Fang H. Peng L. Wei S. Li C. (2019). Comparative transcriptome analysis reveals resistance-related genes and pathways in *Musa acuminata* banana 'Guijiao 9' in response to Fusarium wilt. Plant Physiol. Bioch 141, 83–94. 10.1016/j.plaphy.2019.05.022 31136934

[B57] Swarupa V. Ravishankar K. V. Rekha A. (2014). Plant defense response against *Fusarium oxysporum* and strategies to develop tolerant genotypes in banana. Planta 239, 735–751. 10.1007/s00425-013-2024-8 24420701

[B66] Tian D. Traw M. B. Chen J. Q. Traw M. B. Kreitman M. Bergelson J. (2003). Fitness costs of R-gene-mediated resistance in Arabidopsis thaliana. Nature 423, 74–77. 12721627 10.1038/nature01588

[B58] Uddin A. Mazumder T. H. Barbhuiya P. A. Chakraborty S. (2020). Similarities and dissimilarities of codon usage in mitochondrial ATP genes among fishes, aves, and mammals. IUBMB Life 72, 899–914. 10.1002/iub.2231 31958218

[B59] Wang S. F. Su M. W. Tseng S. P. Li M. C. Tsao C. H. Huang S. W. (2016). Analysis of codon usage preference in hemagglutinin genes of the swine-origin influenza A (H1N1) virus. J. MicrobioImmuno. 49, 477–486. 10.1016/j.jmii.2014.08.011 25442859

[B60] Wani S. H. Anand S. Singh B. Bohra A. Joshi R. (2021). WRKY transcription factors and plant defense responses: latest discoveries and future prospects. Plant Cell Rep. 40 (7), 1071–1085. 10.1007/s00299-021-02691-8 33860345

[B61] Wright F. (1990). The effective number of codons used in a gene. Gene 87, 23–29. 10.1016/0378-1119(90)90491-9 2110097

[B62] Wu Y. Zhao D. Tao J. (2015). Analysis of codon usage patterns in herbaceous Peony (*Paeonia lactiflora* Pall.) based on transcriptome data. Genes 6, 1125–1139. 10.3390/genes6041125 26506393 PMC4690031

[B63] Xin W. Liu Y. Yang Y. Sun T. Niu L. Ge J. (2020). Detection, genetic, and codon usage bias analyses of the VP2 gene of mink bocavirus. Virus Genes 56, 306–315. 10.1007/s11262-020-01738-4 32020392

[B64] Zhang X. Cai Y. Zhai X. Liu J. Zhao W. Ji S. (2018). Comprehensive analysis of codon usage on rabies virus and other lyssaviruses. Int. J. Mol. Sci. 19, 2397. 10.3390/ijms19082397 30110957 PMC6121662

[B65] Zorrilla-Fontanesi Y. Pauwels L. Panis B. Signorelli S. Vanderschuren H. Swennen R. (2020). Strategies to revise agrosystems and breeding to control Fusarium wilt of banana. Nat. Food 1, 599–604. 10.1038/s43016-020-00155-y 37128105

